# An Offset Parameter Optimization Algorithm for Denoising in Photon Counting Lidar

**DOI:** 10.3390/e26110934

**Published:** 2024-10-31

**Authors:** Zhuangbin Tan, Yan Zhang, Ziwen Sun, Jintao Chen, Kun Huang, Yuanjie Qi, Feifan Ma, Zheyu Xu, Renli Zhang, Zhongxing Jiao

**Affiliations:** 1 School of Aeronautics and Astronautics, Sun Yat-sen University, Shenzhen 518107, China; tanzhb6@mail2.sysu.edu.cn (Z.T.); sunzw3@mail2.sysu.edu.cn (Z.S.); chenjt66@mail2.sysu.edu.cn (J.C.); huangk77@mail2.sysu.edu.cn (K.H.); qiyj5@mail2.sysu.edu.cn (Y.Q.); maff@mail2.sysu.edu.cn (F.M.); xuzhy53@mail2.sysu.edu.cn (Z.X.); zhangrli5@mail2.sysu.edu.cn (R.Z.); 2 School of Electronics and Communication Engineering, Sun Yat-sen University, Shenzhen 518107, China; jiaozhx@mail.sysu.edu.cn

**Keywords:** ranging error, solar background noise, MLP network, linear conversion, offset parameter optimization method, anti-noise method

## Abstract

In the case of a weak signal from a photon counting lidar and strong noise from the solar background, the signal is completely submerged by noise, potentially resulting in the appearance of multiple peaks in the denoising algorithm of photon counting entropy. Consequently, a clear distinction between the signal and noise may become challenging, leading to significant fluctuation in the ranging error. To solve this problem, this paper proposes an improved offset parameter optimization algorithm under the framework of photon counting entropy, aiming to effectively eliminate peak interference caused by noise and enhancing ranging accuracy. The algorithm includes two aspects. First, we introduce the solar irradiance prediction of an MLP network and least squares linear conversion to accurately estimate the noise rate of the solar background noise. Then, we propose the offset parameter optimization method to effectively mitigate the interference caused by noise. In simulation and experimental analyses, the ranging error of our proposed method is within 5 and 30 cm, respectively. Compared with the denoising method of photon counting entropy, the average ranging error is increased by 81.99% and 73.76%. Furthermore, compared to other anti-noise methods, it exhibits superior ranging capability.

## 1. Introduction

Photon counting lidar possesses characteristics such as high precision, anti-interference capability, a small size, and a light weight [1]. It can be utilized for long-distance measurement during daytime conditions. Specifically, it can be applied in traffic vehicle detection, drone landing, aircraft landing, disaster and environmental monitoring, highway survey and design, as well as urban design [2,3].

The ranging performance of lidar is influenced by two aspects [4]. First, during the propagation process, the lidar signal undergoes the absorption and scattering by atmospheric molecules and particles [5,6,7,8], resulting in signal attenuation. The lidar signal is also susceptible to interference from solar background noise [9,10] and the dark current [11] of the instrument itself. Solar background noise plays a dominant role. These two aspects weaken the received signal and introduce a significant amount of noise, resulting in a reduction in the signal-to-noise ratio (SNR), which seriously affects the ranging performance of lidar. Therefore, it is necessary to study the denoising algorithms.

Several denoising methods for lidar have been proposed, primarily encompassing three distinct categories, namely, histogram-based, correlation-based, and entropy-based.

First, the histogram-based denoising methods primarily focus on the concentration of the lidar signal and the dispersion of noise [12]. The signal concentration methods include Frank–Wolfe [13,14], proximal gradient [15], and direct thresholding methods [16]. The primary algorithm for noise dispersion is the wavelet threshold method [17]. The two methods are based on the amplitude characteristics of the photon counting histogram and the frequency domain of wavelets. They then employ an effective threshold setting to distinguish between the signal and noise. These algorithms are primarily suitable for dealing with cases where the SNR is high and the signal is significantly stronger than the noise. Under the condition of a low SNR, the noise is relatively strong, and the signal is completely submerged by the noise, which makes these algorithms ineffective.

Then, the correlation-based method, also known as the matched filter method, primarily extracts the signal by performing cross-correlation between the system response and the photon counting histogram. The matched filter method, based on the time-of-flight approach and the time-dependent single-photon-counting technique [18], can effectively mitigate solar background noise in highly scattering environments, thereby enabling more accurate acquisition of depth images for underwater targets. The algorithm is only applicable to cases of high SNR; however, in cases of low SNR, the ranging error is significant [19,20].

Finally, the entropy-based method is more efficient in terms of computational efficiency and effectiveness. Photon counting entropy identifies the target signal through the difference between the signal and noise, based on a correlation analysis of the photon counting histogram image. It has extensive application across diverse domains, such as 3D remote sensing [21], the classification of point clouds from multi-spectral airborne lidar [22], as well as both active and passive remote sensing [23,24]. The photon counting entropy algorithm improves the detection rate even under conditions of low SNR and a weak signal. However, the disadvantage is that random background noise may possess entropy characteristics similar to those of the signal, resulting in issues related to multiple peaks.

The above-mentioned methods have certain limitations. To solve the multi-peak problem of photon counting entropy, we propose an offset parameter optimization algorithm. Our contributions are as follows:

(1) We propose utilizing model prediction and linear conversion to accurately predict the noise rate of the solar background noise, and determine the SNR of the lidar signal, providing prior information for eliminating the influence of solar background noise.

(2) We employ the traversal search with an equal-offset step to optimize the offset parameter, aiming to select an appropriate value that can eliminate the multi-peak interference caused by solar background noise, and improve the ranging performance of lidar.

(3) The validity of photon counting entropy is also analyzed through theoretical derivation for the purpose of verification.

(4) The effectiveness of our proposed denoising algorithm is validated through simulated and measured analysis.

This paper is organized as follows: Section 2 introduces the procedure for generating the simulated signal in photon counting lidar and provides the corresponding analysis of photon counting entropy. Section 3 focuses on describing the framework of our proposed methodology. Section 4 provides our simulation parameters and experimental process. The results and discussion are presented in Section 5. Section 6 gives our conclusions.

## 2. Theoretical Overview

### 2.1. Theoretical Model of Lidar Signal

The mean counts $\bar{y}\left(i\right)$ of photon events (PEs) in the *i*-th time bin, obtained by accumulating *K* pulses, are determined based on Poisson statistics [1,25] for cases with a long dead time. The detected counts y(i) of PEs in the *i*-th time bin exhibit the fluctuation around $\bar{y}\left(i\right)$.

(1) $$\bar{y}\left(i\right)=Kexp\left[-\int_{0}^{(i-1)\Delta t}\lambda_{s}\left(t\right)+\lambda_{n}\left(t\right)dt\right]*\left\{1-exp\left[-\int_{(i-1)\Delta t}^{i\Delta t}\lambda_{s}\left(t\right)+\lambda_{n}\left(t\right)dt\right]\right\},i=1,\ldots ,N$$

where $\lambda_{s}$(t) is the received signal rate; and $\lambda_{n}\left(t\right)$ denotes the total noise rate caused by solar background noise and dark current. The noise in lidar daytime detection primarily originates from solar background noise, while the impact of dark current is negligible [1]; Δt represents the resolution of the time correlator; and *N* is the number of time bins.

For a typical flat target with slope and roughness [26,27], it is believed that $\lambda_{s}\left(t\right)$ follows a Gaussian distribution, as indicated by Equation (2). According to Equation, the primary factors influencing $\lambda_{s}\left(t\right)$ are transmission transmittance (τ) and propagation time (*t*). The accurate theoretical calculation of τ can be found in Section 2.2.

(2) $$\lambda_{s}\left(t\right)=N_{s}\frac{1}{\sqrt{2\pi }\sigma_{s}}exp\left(-\frac{t^{2}}{2\sigma_{s}^{2}}\right)=\frac{\eta_{q}\eta_{r}E_{t}A_{r}\tau^{2}\beta_{r}cos\theta_{g}}{\pi hvH^{2}}\frac{1}{\sqrt{2\pi }\sigma_{s}}exp\left(-\frac{t^{2}}{2\sigma_{s}^{2}}\right)$$

where the width of the received lidar pulse is denoted as $\sigma_{s}$; $\eta_{q}$ represents the quantum efficiency of the detector; $\eta_{r}$ represents the efficiency of the receiver optics; $E_{t}$ is the energy of the transmitted laser; τ is defined as the transmittance of the laser. The aperture of the receiving telescope is denoted as $A_{r}$; $\beta_{r}$ represents the intensity reflection coefficient of the ground target; $\theta_{g}$ is the intersection angle between the ray axis and the normal of the target surface; and *h*, *v*, and *H* represent the Planck constant, laser frequency, and propagation distance, respectively.

The noise rate $\lambda_{n}\left(t\right)$ is used to evaluate the level of noise caused by global horizontal irradiance (GHI). The following two stages are proposed to accurately estimate $\lambda_{n}\left(t\right)$. First, the GHI is predicted by the MLP network; see Section 2.3 for further details. $\lambda_{n}\left(t\right)$ can be estimated by linearly converting GHI into $\lambda_{n}\left(t\right)$, as outlined in Section 3.1.

### 2.2. Theoretical Model of Laser Transmission

According to the Beer–Lambert–Bouguer law [5], the transmission transmittance τ of lidar in the atmosphere is accurately calculated by Equation (3).

(3) $$\tau =exp^{-\int_{0}^{H}k_{m}\left(h\right)+k_{n}\left(h\right)+\beta_{m}\left(h\right)+\beta_{n}\left(h\right)\;dh}$$

where $k_{m}$ and $\beta_{m}$ represent the absorption and scattering coefficients of atmospheric molecules, respectively; and $k_{n}$ and $\beta_{n}$ are the absorption and scattering coefficients of particles.

The line-by-line integration method is utilized for analyzing $k_{m}$ [7,28]. $\beta_{m}$ is calculated from the Rayleigh scattering [29]. $k_{n}$ and $\beta_{n}$ are analyzed using Mie scattering [30,31] by Equation (4).

(4) $$k_{n}+\beta_{n}=\int_{r_{1}}^{r_{2}}\left(C_{sca}+C_{abs}\right)F\left(r\right)\;dr$$

where $C_{abs}$ and $C_{sca}$ represent the cross-sections for absorption and scattering, respectively; *r* is the particle radius. The aerosol size distribution F(r) is modeled using the gamma distribution in Equation (5).

(5) $$F\left(r\right)=ar^{c}exp^{-br^{d}}$$

where *a*, *b*, *c*, *d* represent coefficients of size distribution, with the specific values provided in Table 1.

The above methods are used to calculate τ under different weather conditions of rain and fog in Table 1, as well as varying transmission distances. The corresponding results are shown in Table 2.

### 2.3. GHI Prediction of MLP Model

**Figure 1:**
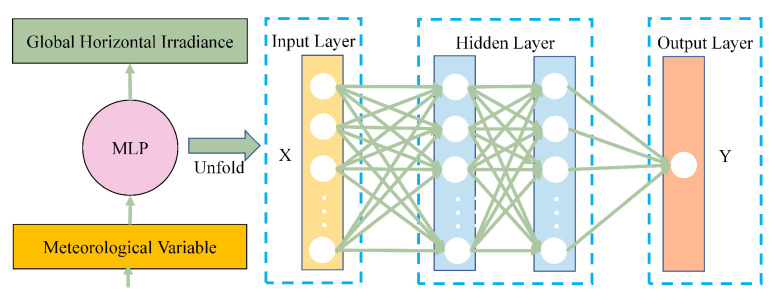
The structure of multi-layer perceptron model.

The GHI is accurately predicted using the MLP network [32]. It consists of a hierarchical arrangement of interconnected nodes, also known as neurons, which are organized into an input layer, four hidden layers, and an output layer. The details are illustrated in Figure 1.

**Figure 2:**
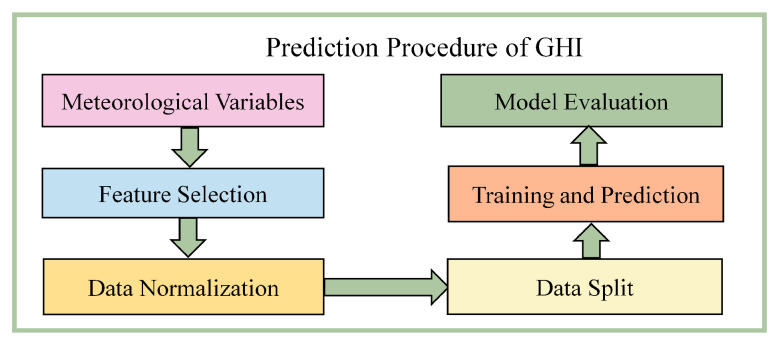
Procedure of predicting GHI.

The MLP network is primarily used to establish the complex correlation between GHI and meteorological variables. The main procedures for predicting GHI, as shown in Figure 2, include the input of meteorological variables, feature selection, data pre-processing, training and prediction of the model, and model evaluation.

Our study utilizes a two-stage feature optimization algorithm to identify the optimal subset of meteorological variables. The feature optimization process involves using the correlation coefficient [33] to determine the most relevant meteorological variables for GHI, and utilizing a recursive feature analysis framework based on the variance inflation factor [34,35] to eliminate redundant terms among these variables.

The normalization [36] effectively narrows the value range of various meteorological variables, thereby mitigating the excessive impact of certain variables on model training. Meanwhile, it can enhance the convergence speed and overall robustness of the model. The normalization process can be divided into two distinct stages: The initial stage involves determining the maximum ($X_{max}$) and minimum ($X_{min}$) values for each meteorological variable, while the subsequent stage involves normalizing the value $X_{nor}$ of each variable based on Equation (6).

(6) $$X_{nor}=\frac{X-X_{min}}{X_{max}-X_{min}}$$

The optimal subset of meteorological variables, obtained through the feature optimization algorithm, is integrated with GHI to facilitate training and prediction procedures in the MLP network. The data are primarily divided into two segments: the initial 80% constitutes the training set, while the remaining 20% forms the test set. The training set is primarily used for network calibration, while the test set is employed to evaluate the performance of the network.

The standardized hyperparameters obtained from corresponding benchmarks are used to construct the MLP model. The optimal values are obtained through training and prediction of the model, which is performed at least five times. The values of the hyperparameters are listed in Table 3.

The accuracy of the GHI prediction can be effectively evaluated using various statistical metrics, including root mean square error (RMSE) [37], relative root mean square error (RRMSE) [38], mean absolute error (MAE) [39,40], ratio of the root mean square error to the standard deviation of measurement data (RSR) [38], Willmott’s index (WI) [41], and the coefficient of determination (R2) [33]. For better model prediction performance, RMSE, RRMSE, MAE, and RSR should be closer to 0.0, while R2 and WI should approach 1.0 as closely as possible. The corresponding mathematical expressions are as follows:

(7) $$RMSE=\sqrt{\frac{1}{n}\sum_{i=1}^{n}{\left(Y_{m}-Y_{p}\right)}^{2}}$$

(8) $$RRMSE=\frac{\sqrt{\frac{1}{n}\sum_{i=1}^{n}{\left(Y_{m}-Y_{p}\right)}^{2}}}{\bar{Y}}$$

(9) $$MAE=\frac{1}{n}\sum_{i=1}^{n}\left|Y_{m}-Y_{p}\right|$$

(10) $$RSR=\frac{\sqrt{\sum_{i=1}^{n}{\left(Y_{m}-Y_{p}\right)}^{2}}}{\sqrt{\sum_{i=1}^{n}{\left(Y_{m}-\bar{Y}\right)}^{2}}}$$

(11) $$WI=1-\frac{\sum_{i=1}^{n}{\left(Y_{m}-Y_{p}\right)}^{2}}{\sum_{i=1}^{n}\left(\right|Y_{p}-\bar{Y}\left|+\right|Y_{m}-\bar{Y}{\left|\right)}^{2}}$$

(12) $$R2=1-\frac{\sum {\left(Y_{m}-Y_{p}\right)}^{2}}{\sum {\left(Y_{m}-\bar{Y}\right)}^{2}}$$

where $Y_{m}$, $Y_{p}$, and $\bar{Y}$ are the actual, predicted, and actual mean values of the GHI, respectively. The units of both MAE and RMSE are $W/m^{2}$.

### 2.4. The Analysis of Photon Counting Entropy

The photon counting entropy can be utilized for assessing the inherent uncertainty of random events and quantifying the uncertainty in photon events in the response of GM-APD. In the detection process of single-photon-counting lidar, the solar background noise appears as a stationary random process, while the signal is a non-stationary random process. Therefore, the uncertainty of photon events in solar background noise is significantly higher than that of the photon events in signal.

The estimated random fluctuation $\hat{y}\left(i\right)$ of the detected counts in the *i*-th time bin can be calculated using Equation (13) based on the noise rate ${\hat{\lambda }}_{n}\left(t\right)$ of solar background noise acquired through prediction of the MLP model and linear conversion.

(13) $$\hat{y}\left(i\right)=y\left(i\right)-Kexp\left[-\int_{0}^{(i-1)\Delta t}{\hat{\lambda }}_{n}\left(t\right)\right]*\left(1-exp\left[-\int_{(i-1)\Delta t}^{i\Delta t}{\hat{\lambda }}_{n}\left(t\right)dt\right]\right)$$

The discrete Fourier transform of $\hat{y}\left(i\right)$ is obtained using Equation (14), while the corresponding photon counting entropy Hc [1] is calculated using Equation (15).

(14) $$\hat{Y}\left(k\right)=\sum_{i=1}^{N}\hat{y}\left(i\right)exp\left(-\frac{2\pi kij}{N_{k}}\right)$$

(15) $$Hc=-\sum_{k=1}^{N_{k}}\frac{|\hat{Y}{\left(k\right)|}^{2}}{\sum_{k=1}^{N_{k}}{\left|\hat{Y}\left(k\right)\right|}^{2}}ln\frac{|\hat{Y}{\left(k\right)|}^{2}}{\sum_{k=1}^{N_{k}}{\left|\hat{Y}\left(k\right)\right|}^{2}}$$

where $\hat{Y}\left(k\right)$, $k=1,2,\ldots ,N_{k}$ represents the *k*-th sampling point in the Fourier domain of $\hat{y}\left(i\right)$, i=1,2,…,N, and $N_{k}$ denotes the number of discrete frequency points. $|\hat{Y}\left(k\right)|$ represents the power of the *k*-th discrete frequency point.

**Figure 3:**
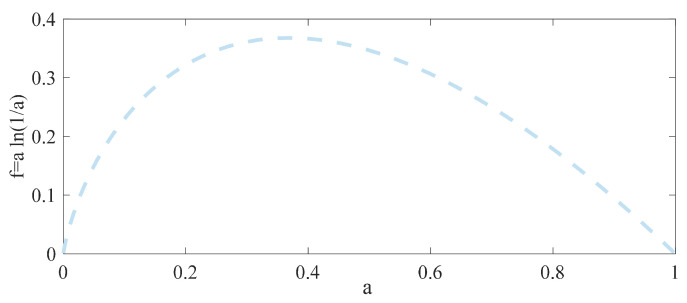
The characteristics of photon counting entropy.

The characteristics of photon counting entropy are preliminarily analyzed through simulations using $a=\frac{|\hat{Y}{\left(k\right)|}^{2}}{\sum_{k=1}^{N_{k}}{\left|\hat{Y}\left(k\right)\right|}^{2}}$ and f=aln(a), as shown in Figure 3. The figure illustrates that each term of the photon counting entropy follows a convex pattern, where its average value is lower on both sides compared to the middle. The noise remains stable under the discrete Fourier transform, while the signal exhibits fluctuating behavior. This suggests that the signal fluctuates around the noise, indicating a significantly higher photon counting entropy for the noise compared to the signal. A more comprehensive verification is provided in the Appendix A.

The photon counting entropy can quantify the uncertainty caused by both noise and signal to some extent, thereby reducing the impact of solar background noise and enhancing the accuracy of lidar ranging. However, due to the increase in solar background noise, the problem of multiple peaks arises, making it difficult to accurately determine the existence and location of signals.

## 3. The Proposed Methodology

**Figure 4:**
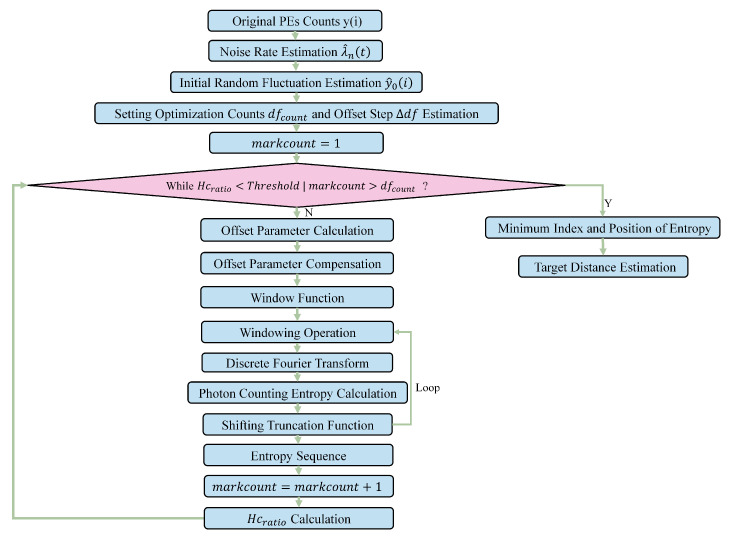
The workflow of our proposed method.

The presence of multiple peaks in photon counting entropy, caused by solar background noise, poses challenges in determining the peak position of the target signal and enhancing the ranging accuracy for photon counting lidar. To address this issue, we propose an offset parameter df optimization algorithm under the framework of photon counting entropy. The proposed algorithm combines the accurate estimation of the noise rate ${\hat{\lambda }}_{n}\left(t\right)$ and optimization of df to mitigate the influence of solar background noise. The overall process is illustrated in Figure 4.

### 3.1. Estimating Noise Rate

When the detector receives the echo signal, the original counts y(i) of the corresponding photon events (PEs) in the *i*-th time bin are obtained. It is essential to analyze the level of solar background noise and accurately estimate the corresponding noise rate ${\hat{\lambda }}_{n}\left(t\right)$. Its estimation consists of two stages: The initial stage involves constructing the MLP network to predict GHI by training meteorological variables, as outlined in Section 2.3; then, the ${\hat{\lambda }}_{n}\left(t\right)$ is estimated by evaluating the correlation between it and the GHI.

The correlation conversion between GHI and ${\hat{\lambda }}_{n}\left(t\right)$ primarily considers two aspects: First, the single-photon-counting lidar demonstrates a potential proportional relationship between the GHI across the entire wavelength range and that of each individual wavelength. The second factor involves converting the proportional correlation between GHI and ${\hat{\lambda }}_{n}\left(t\right)$ at a specific wavelength. Therefore, their relationship can be considered a linear correlation, which is described by Equation (16).

(16) $${\hat{\lambda }}_{n}\left(t\right)=C_{1}*\mathrm{GHI}+C_{2}$$

where $C_{1}$ and $C_{2}$ are the fitting coefficients.

In practice, the model can be pre-trained, and only a small dataset is needed to accurately estimate the noise rate ${\hat{\lambda }}_{n}\left(t\right)$. Therefore, the calculation efficiency of the algorithm is minimally affected by the model.

### 3.2. Initial Random Fluctuation Estimation

Based on the estimated ${\hat{\lambda }}_{n}\left(t\right)$, Equation (13) is utilized for subtracting the mean noise of the Poisson distribution from the original counts y(i), further obtaining the initial random fluctuation ${\hat{y}}_{0}\left(i\right)$. However, there still exists significant noise jitter in ${\hat{y}}_{0}\left(i\right)$, which can affect the extraction of the photon counting entropy.

### 3.3. Offset Parameter Optimization and Compensation

To further reduce the impact of solar background noise, we consider subtracting a specific offset parameter df from the ${\hat{y}}_{0}\left(i\right)$. But, the selection of df has a significant impact on the improvement of the SNR. Therefore, we propose the df optimization algorithm to conduct the traversal search with an equal-offset step Δdf, and then, select the appropriate one.

Maximum and minimum values of ${\hat{y}}_{0}\left(i\right),i=1,\ldots ,N$ can be employed for determining the upper $df_{upper}$ and lower $df_{lower}$ bounds of optimizing df. By selecting the maximum number $df_{count}$ of optimization iterations, the offset step Δdf can be calculated by Equation (17).

(17) $$\Delta df=\frac{df_{upper}-df_{lower}}{df_{count}}$$

In each iteration of the optimization process, the df is calculated by Equation (18), and then, compensated to the ${\hat{y}}_{0}\left(i\right)$ by Equation (19), further obtaining the current random fluctuation $\hat{y}\left(i\right)$.

(18) $$df=df_{lower}+markcount\Delta df$$

(19) $$\hat{y}\left(i\right)={\hat{y}}_{0}\left(i\right)-df,i=1,\ldots N$$

where markcount ($markcount=1,2,\ldots ,df_{count}$) represents the markcount-th iteration.

### 3.4. Window Function

To divide the sampling period into several subintervals and calculate the photon counting entropy for each subinterval, the Hamming window is used [1] in this study, with its corresponding expression given by Equation (20).

(20) $$W\left(m\right)=\left\{\begin{array}{cc} 0.54-0.46cos\frac{2\pi m}{M-1}, & 0\leq m\leq M-1\\ 0.0, & \mathrm{else} \end{array}\right.$$

where *M* is the window width, generally $M=6.5\sigma_{s}$.

### 3.5. Noise Mitigation

By incorporating the window function, the sampling period of the signal can be divided into *Q* subintervals. After obtaining the current $\hat{y}\left(i\right)$, the corresponding random fluctuation ${\hat{y}}_{q}\left(m\right)$ for each subinterval can be calculated by Equation (21).

(21) $${\hat{y}}_{q}\left(m\right)=W\left(m\right)\hat{y}\left(m+\left(q-1\right)s\right),m=0,\ldots M-1$$

where *s* is the interval between two adjacent window operations; generally, s = 1 time bin. q,q=1,…,Q represents the *q*-th subinterval; and *Q* is the number of subintervals, Q=(N−M−s)/s.

The random fluctuation ${\hat{y}}_{q}\left(m\right)$ of the *q*-th subinterval is analyzed using discrete Fourier transform, and then, the corresponding photon counting entropy is $Hc_{q}$.

(22) $${\hat{Y}}_{q}\left(k\right)=\sum_{m=1}^{M}{\hat{y}}_{q}\left(m\right)exp\left(-j2\pi km/N_{k}\right)$$

(23) $$Hc_{q}=-\sum_{k=1}^{N_{k}}\frac{|{\hat{Y}}_{q}{\left(k\right)|}^{2}}{\sum_{k=1}^{N_{k}}{\left|{\hat{Y}}_{q}\left(k\right)\right|}^{2}}ln\frac{|{\hat{Y}}_{q}{\left(k\right)|}^{2}}{\sum_{k=1}^{N_{k}}{\left|{\hat{Y}}_{q}\left(k\right)\right|}^{2}}$$

where ${\hat{Y}}_{q}\left(k\right),k=1,2,\ldots ,N_{k}$ represents the *k*-th sampling point in the Fourier domain; and $N_{k}$ is the number of the discrete frequency point. With the shifting of the window function, the sequence of the photon counting entropy is shown by Equation (24). Meanwhile, replacing the raw counts y(i) with the sequence of the photon counting entropy can further mitigate the effect of solar background noise.

(24) $$Hc=[Hc_{1},Hc_{2},\ldots ,Hc_{Q}]$$

### 3.6. Extreme Ratio of Photon Counting Entropy

To address the problem of threshold determination during optimization of the offset parameter df, we propose the extreme ratio $Hc_{ratio}$ of the photon counting entropy. The $Hc_{ratio}$ primarily represents the relative difference between the minimum and second smallest values of the photon counting entropy, in order to preserve only one peak caused by the signal in a sequence of the photon counting entropy.

(25) $$Hc_{ratio}=\frac{Hc_{max}-Hc_{secmin}}{Hc_{max}-Hc_{min}}$$

where $Hc_{max}$, $Hc_{min}$, and $Hc_{secmin}$ represent the maximum, minimum, and the second smallest values of Hc, respectively.

Meanwhile, we set a corresponding Threshold, initially at 0.5. When the extreme ratio $Hc_{ratio}$ is lower than Threshold, the optimization process is completed and a suitable df is obtained. If the maximum number ($df_{count}$) of iterations is reached, the df corresponding to the minimum $Hc_{ratio}$ is selected as the result.

### 3.7. Range Estimation

After completing the df optimization, the sequence Hc of the photon counting entropy corresponding to the minimum $Hc_{ratio}$ in the iterative optimization is selected for analysis. The propagation time $t_{q0}$ of the target signal corresponds to the time delay of the q0-th subinterval, where the photon counting entropy is minimal.

(26) $$t_{q_{0}}=\frac{M}{2}\Delta t+\left(q_{0}-1\right)s\Delta t$$

The target distance *R* can be estimated as follows:

(27) $$R=\frac{1}{2}ct_{q_{0}}$$

### 3.8. Ranging Performance Evaluation

The estimation of ranging error is crucial for validating the performance of our proposed algorithm, and the corresponding equation [1] is presented below.

(28) $$\sigma_{error}=\frac{1}{P}\sum_{p=1}^{P}\left|R_{s,p}-R_{true,p}\right|$$

where $R_{true,p}$ represents the precise distance to the target in the *p*-th measurement; $R_{s,p}$ is the distance of the *p*-th measurement estimated by our proposed algorithm; and *P* is the total number of experimental measurements.

To evaluate the improvement of our proposed method in ranging error compared to the other algorithm, we primarily utilize the following formula:

(29) $$IM=\frac{1}{P}\sum_{p=1}^{P}\frac{|R_{o,p}-T_{true,p}\left|-\right|R_{s,p}-R_{true,p}|}{|R_{o,p}-R_{true,p}|}$$

where $R_{true,p}$, $R_{o,p}$, and $R_{s,p}$ represent the actual target distance, and the target distances calculated by the other algorithm and our proposed method in the *p*-th detection, respectively; and *P* is the total number of experimental measurements.

## 4. Simulation and Experiment

### 4.1. Experimental Setup of Simulation

The response characteristics of Gm-APD are simulated in this study using the Monte Carlo method. The simulation is conducted in the Matlab R2021b environment using a professional scientific research license, ensuring computational accuracy and consistency of the results. The effectiveness of our proposed algorithm is validated through the evaluation of the ranging error. The specific simulation settings are primarily derived from Equations (1)–(3), with the corresponding common simulation parameters presented in Table 4. The noise rate can be estimated by referring to Section 4.2.

### 4.2. The Measurement of Background Noise Rate

**Figure 5:**
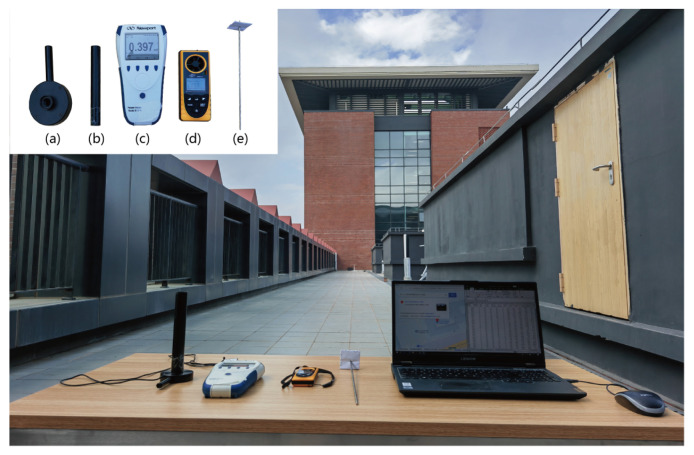
Experimental setup for measuring outdoor solar background irradiance.

To accurately predict the GHI and estimate the corresponding ${\hat{\lambda }}_{n}\left(t\right)$, we conducted relevant outdoor experiments at Sun Yat-sen University Shenzhen Campus in Guangming District, Shenzhen, China, as shown in Figure 5. Our experimental setup included the 843-R-USB power meter, 919P-003-10 thermopile sensor (namely, lens), the Benetech GM8910 anemometer, a black opaque cylinder, a piece of paper, and our computer, as depicted in Figure 5a–d. The 843-R-USB power meter in Figure 5c will be paired with the lens in Figure 5a to observe the solar background irradiance. The detection capability of the power meter heavily depends on both the measurement intensity and the bandwidth range of the thermopile sensor. The black opaque cylinder in Figure 5b is primarily utilized for receiving the direct sunlight and blocking the sunlight in other directions. The paper in Figure 5e is used to block the direct sunlight. The Benetech GM8910 anemometer in Figure 5d can be used for measuring meteorological variables. The technical specifications of the power meter, 919P-003-10 thermopile sensor and Benetech GM8910 anemometer are listed in Table 5, Table 6 and Table 7, respectively. These instruments are utilized to measure solar background irradiance and meteorological variables, as described in Table 8. Our experiments aim to predict GHI during the daytime (from 8 am to 6 pm) under both cloudy and sunny weather conditions, as there is no irradiance at night.

The variables were measured at 5 min intervals throughout the specific observation process. The details are as follows: (1) The lens aperture of the power meter is directed towards the sun to capture sunlight from all directions, and a reading of the power meter is taken and recorded as the GHI. (2) The cylinder is inserted into the lens aperture, precisely oriented towards the sunlight, and a reading is taken with the power meter to record it as the DNI. (3) We remove the cylinder, align the lens aperture directly towards the sun, obstruct direct sunlight with a piece of paper to enable reception of light from other directions, and subsequently, record the reading of the power meter as the DHI; (4) T, RH, DP, P, and A can be obtained by pressing the buttons on the anemometer in sequence. (5) The anemometer is set to the speed option, with the fan blades aligned towards east and north, while recording the corresponding readings as MWS and LWS, respectively.

**Figure 6:**
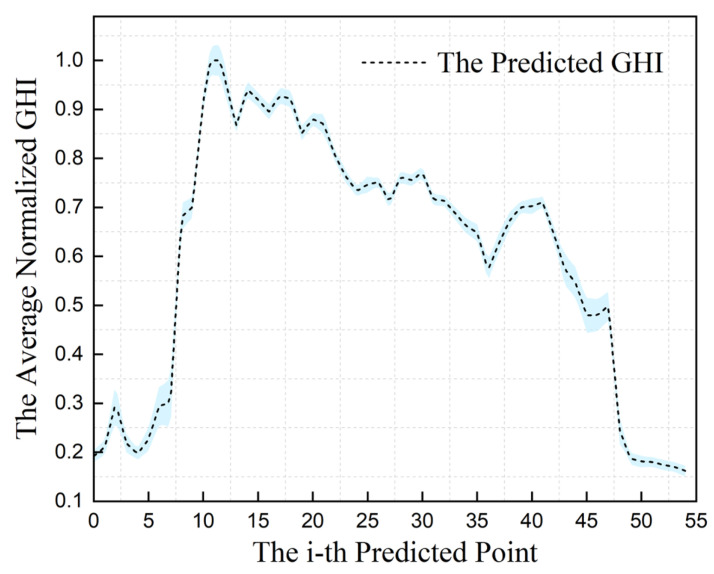
The error bar for the standard deviation of GHI prediction.

The characteristics of the GHI obtained from the experiments are analyzed in Table 9. The optimal meteorological variables optimized by the two-stage feature optimization algorithm based on the correlation and redundancy between variables are DNI, DHI, T, RH, and LWS. These variables were integrated into the MLP network for GHI prediction. The MLP model was employed for 20 iterations of GHI training and prediction, yielding error bars for the standard deviation of GHI prediction as depicted in Figure 6. The mean model metrics for RMSE, RRMSE, MAE, RSR, WI, and R2 were 0.0821, 0.1323, 0.0571, 0.2900, 0.9787, and 0.9171, respectively.

**Figure 7:**
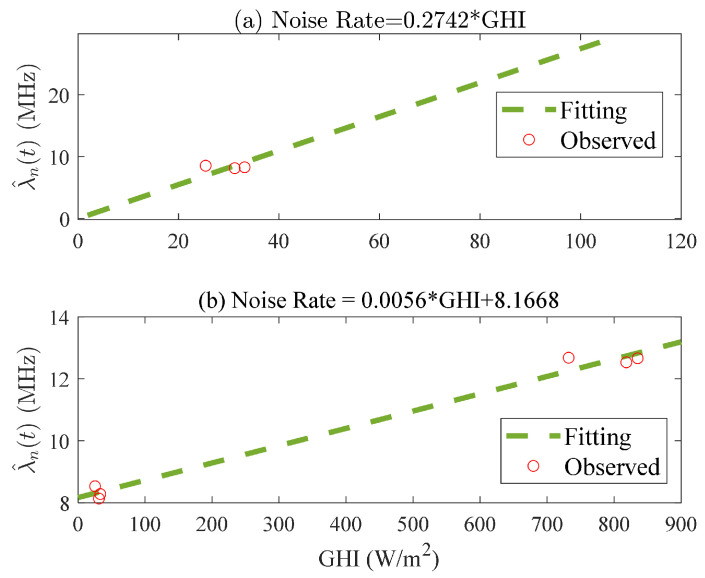
The linear relationship between GHI and ${\hat{\lambda }}_{n}\left(t\right)$ using the measurement data.

The linear conversion from GHI to ${\hat{\lambda }}_{n}\left(t\right)$ is precisely achieved through six sets of corresponding experiments. For the analysis of the simulation experiments, we consider increasing the ${\hat{\lambda }}_{n}\left(t\right)$ as much as possible. The first three sets of measurement data are selected for fitting using the least squares method. The result corresponding to Figure 7a is presented. The six pairs of data are used for fitting in order to accurately analyze the measured data, and the corresponding results are presented in Figure 7b.

### 4.3. The Ranging Experiment

**Figure 8:**
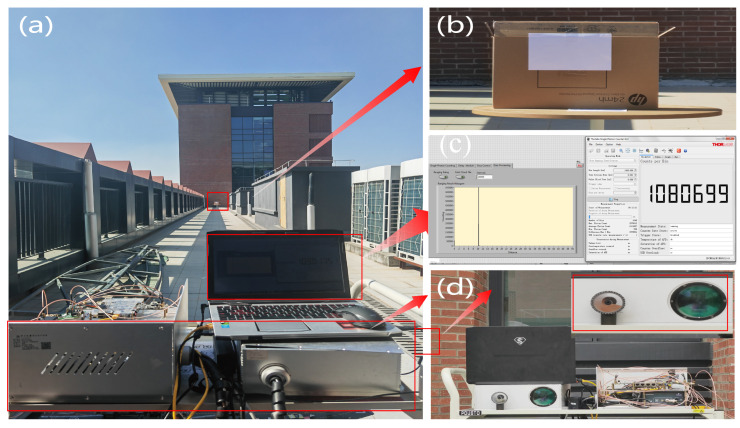
The photongraph of the experimental setup. (**a**) The experiment scene. (**b**) The target (A4 paper) used in the experiment. (**c**) The photon counting histogram of the received signal (**d**) The attenuation lens used for reducing the intensity of the transmitted signal.

The relevant ranging experiments for single-photon-counting lidar were conducted at the same location. The experimental scenes and equipment are shown in Figure 8, while the corresponding parameters are taken from Table 4. The experimental setup primarily consists of a computer in Figure 8a,c, a lidar system in Figure 8a, an empty box with a target (specifically A4 paper) in Figure 8b, and an attenuation lens in Figure 8d. The computer is primarily used for storing experimental data and visualizing the distribution of the received signal through a histogram. The attenuation lens is placed at the transmitting port of the lidar system to reduce the intensity of the transmitted signal. The lidar system utilizes pulsed laser for precise targeting and primarily consists of a PIN detector, a Si SPAD detector, and a time-correlated single-photon-counting (TCSPC) module. The PIN detector (DET025 A/M) is equipped with a voltage amplifier that emits laser pulses synchronously, thereby generating a start signal for the TCSPC module (TDCGP22). Then, the Si SPAD (SPCM50 A/M) detects and amplifies the echo signal captured by the receiving module, thus triggering an avalanche response. The TCSPC module accurately records the precise arrival time of each photon and generates a histogram based on these recorded events.

Our single-photon-counting lidar was initially designed to accurately measure target distances of thousands of meters, with a precision of 1 cm. However, in fact, due to factors such as imaging focusing and others, we chose to conduct experiments at distances of tens of meters. Under these experimental conditions, the only difference between our experiments and experiments conducted at several thousand meters is the SNR. The measurement accuracy of the lidar and the recognition accuracy of our proposed algorithm remain unchanged. To meet the requirements of experiments, we intentionally degrade the SNR by increasing the noise level and reducing the signal strength in the experiments, aiming to simulate long-distance measurements under specific conditions. At this time, the single-photon-counting lidar may not be capable of directly extracting the target from the original signal or achieving accurate identification due to its low SNR.

The SNR in our experiments was primarily affected by three factors, namely, the target distance, attenuation angle, and the accumulated number of laser pulses. The calibration experiments were primarily conducted in two sequential steps.

Step 1. The meteorological variables, as shown in Figure 5, were initially measured using the power meter and anemometer before conducting lidar experiments at each calibration distance to estimate the noise rate ${\hat{\lambda }}_{n}\left(t\right)$. The attenuation lens was initially calibrated at an angle of 160 deg, and subsequently, rotated downward by 30 deg each time. The lidar signal underwent a series of five successive adjustments (160, 130, 100, 70, and 40 deg) to gradually decrease its intensity. The lidar emitted 200,000 pulses for each angle, while the receiver detected approximately half as many effective pulse events.

Step 2. The calibration distances were measured for 7 groups, specifically at 31.4, 37.5, 43.7, 47.7, 53.4, 58.4, and 64.1 m. The procedure described in step 1 was repeated for each group.

## 5. Results and Discussion

The laser ranging scheme primarily focuses on extracting the weak signal and reducing the strong solar background noise in the lidar echo signal, especially under low SNR conditions. The calculation of the SNR [1,17] can be performed using Equation (30). The effectiveness of the proposed algorithm is validated through the analysis of simulation and measured data.

(30) $$SNR=\frac{\sqrt{K}N_{s}}{\sqrt{N_{s}+N_{n}}}$$

where $N_{n}$ represents the number of photoelectrons generated by noise, which is further calculated by Equation (31).

(31) $$N_{n}=\Delta t\lambda_{n}N$$

where $\lambda_{n}$ represents the real noise rate of the solar background noise.

### 5.1. Simulation Analysis

**Figure 9:**
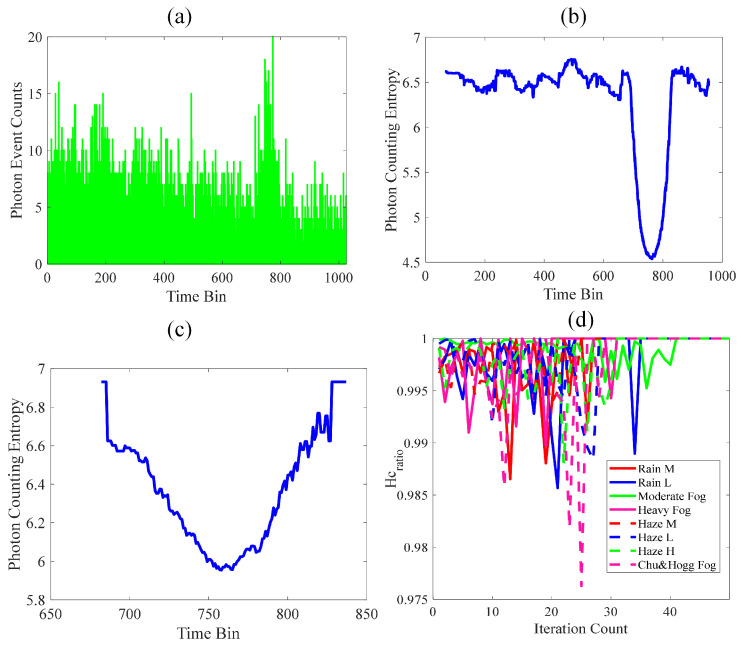
The simulation analysis of our proposed method. (**a**) Photon counting histogram for heavy fog. (**b**) The photon counting entropy obtained without optimization of the offset parameter. (**c**) The photon counting entropy obtained with optimization. (**d**) The threshold determination of $Hc_{ratio}$.

For the preliminary simulation test of our proposed method, the parameters selected were as follows: The target distance was set to 500 m, corresponding to the position of the 760-th time bin; the ${\hat{\lambda }}_{n}\left(t\right)$ was 14.1 MHz and eight different size distributions were chosen. The analytical outcomes of the PEs and photon counting entropy of Gm-APD in lidar are illustrated in Figure 9.

The signal and noise in Figure 9a are observed to be mixed, making it challenging to distinguish between them. The photon counting entropy in Figure 9b is obtained without optimizing the offset parameter (df=0), while Figure 9c illustrates the photon counting entropy achieved after optimization (df=2.4). The two obtained results can clearly differentiate between the signal and noise. Furthermore, our proposed method enables selection retention of the peak portion of the signal in photon counting entropy while eliminating peaks caused by solar background noise, thereby improving the accuracy of signal localization. The threshold for optimizing the $H_{c\_ratio}$ to its optimum is determined by analyzing the variation in $H_{c\_ratio}$ during the iterative optimization of df under different size distributions, as illustrated in Figure 9d. The majority of them fall within the range of 0.985 to 1.0, indicating that the initial threshold of 0.985 is appropriate.

In the simulation analysis, the simulation parameters were mainly selected based on the key factors that affect the SNR of the lidar signal. According to Equation (30), the main factors affecting the SNR are *K*, $N_{n}$, and $N_{s}$. Based on Equations (2) and (31), it can be further deduced that the primary factors affecting $N_{n}$ and $N_{s}$ within the given lidar detection system are $\lambda_{n}$ and *H*. Therefore, we compared the raging error obtained by various methods from three factors, namely, the noise rate ${\hat{\lambda }}_{n}\left(t\right)$ of the solar background noise, the accumulated number *K* of laser pulses, and the target distance *H*, in order to validate the performance of the proposed method. These methods include our proposed method, photon counting entropy, and the matched filter, direct thresholding, Frank–Wolfe, proximal gradient, and wavelet threshold methods.

**Figure 10:**
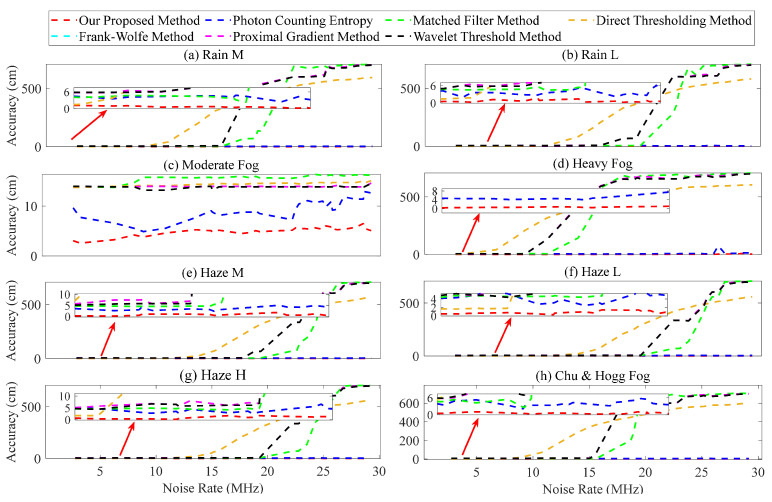
Ranging error of the different estimated noise rates.

In terms of the noise rate ${\hat{\lambda }}_{n}\left(t\right)$, the following simulation parameters were selected: The observed GHI was considered as the truth, while the GHI obtained from the prediction of the MLP network was regarded as the estimated value; their values ranged from 10 to 104 $W/m^{2}$; the corresponding true $\lambda_{n}\left(t\right)$ and estimated ${\hat{\lambda }}_{n}\left(t\right)$ values, obtained through the linear conversion in Figure 7a, ranged from 2.7 to 29.6 MHz; for the different size distributions, namely, rain M, rain L, heavy fog, haze M, haze L, haze H, and Chu and Hogg fog, their respective target distances were set at 500 m; however, due to the significantly reduced transmittance of moderate fog at a distance of 500 m, it was adjusted to 100 m; the number of laser pulses was 10,000; the range of the SNR was between 4.7 and 35.9. The average ranging error obtained from 10 Monte Carlo simulation measurements is shown in Figure 10.

The graph illustrates the gradual increase in ranging error as the noise rate increases. The results depicted in Figure 10a,b,d–h demonstrate that for ${\hat{\lambda }}_{n}\left(t\right)$ below 15 MHz, the matched filter, direct thresholding, Frank–Wolfe, proximal gradient, and wavelet threshold methods exhibit a small error, within 10 cm. However, the ranging error of these methods increases significantly when ${\hat{\lambda }}_{n}\left(t\right)$ exceeds 15 MHz, reaching over 600 cm. The moderate fog in Figure 10c exhibits satisfactory performance across various methods, with a ranging error of less than 20 cm, which can be attributed to the high SNR related to a target distance of 100 m. The photon counting entropy and our proposed method effectively quantify the uncertainty between the signal and noise, enabling precise determination of the signal’s position while ensuring that the ranging error remains below 15 cm, as demonstrated in Figure 10. The proposed method performs better, with an error of approximately 5 cm.

**Figure 11:**
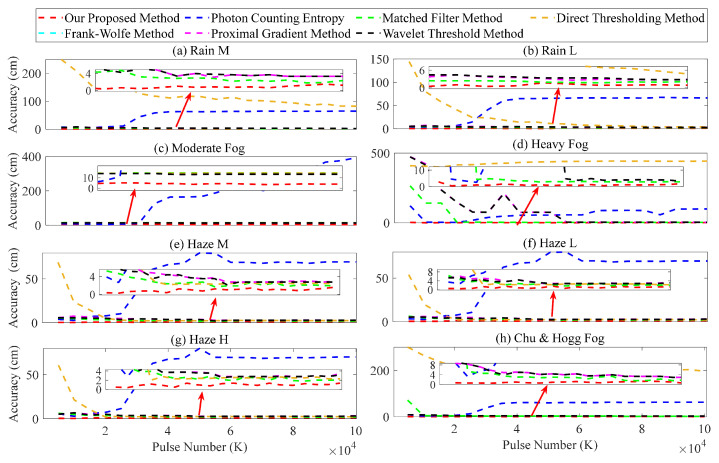
Ranging error in the different accumulated number of laser pulses.

Based on the accumulated number *K* of laser pulses, we also evaluate the ranging performance of various methods. The simulation parameters selected are as follows: $\lambda_{n}\left(t\right)$ and ${\hat{\lambda }}_{n}\left(t\right)$ are 13.7 and 14.1 MHz, respectively; *K* ranges from $0.5\times 10^{4}$ to $10\times 10^{4}$, with an interval of $0.55\times 10^{4}$; the SNR varies between 4.8 and 68.2; the process of selecting other parameters is similar to analyzing the impact of the noise rate. The average error outcomes obtained from 10 simulation calculations are shown in Figure 11.

The analyses of the Frank–Wolfe and proximal gradient methods and our proposed method in Figure 11 demonstrate that the ranging error is minimally influenced by *K*. The Frank–Wolfe and proximal gradient methods demonstrate satisfactory performance, with the error within 10 cm, while our proposed method exhibits superior performance, with an error of approximately 5 cm. The results illustrated in Figure 11d,h indicate that when *K* falls below a certain threshold, such as $1\times 10^{4}$, both the matched filter and wavelet threshold methods exhibit significant fluctuations and errors, reaching over 100 cm. As observed in Figure 11a,b,d–h, the direct thresholding method also exhibits similar fluctuations, resulting in errors exceeding 200 cm. Furthermore, Figure 11a–c,e–h demonstrates the gradual increase in ranging error as *K* increases when employing the method of photon counting entropy; this may exceed 50 cm.

**Figure 12:**
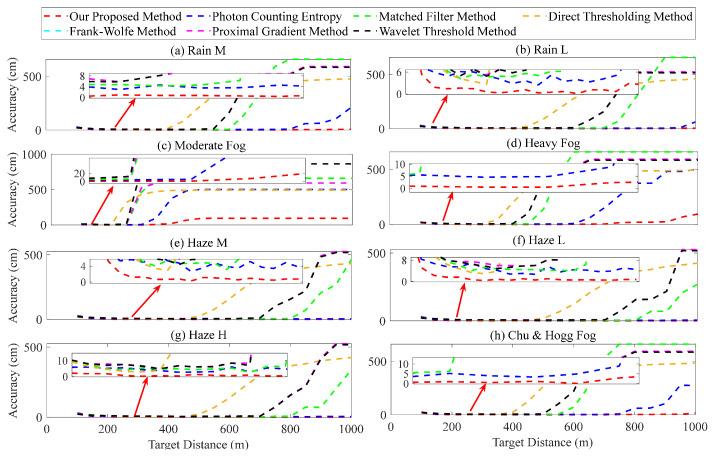
Ranging error for different target distances.

The evaluation of the ranging performance of various methods was conducted simultaneously, taking into account the target distance *H*. The simulation parameters were as follows: *K* was set at $1\times 10^{4}$; the target distances ranged from 100 to 1000 m, with an interval of 50 m; the SNR ranged from 0.01 to 225.08; the selection process for other parameters was based on an analysis of the accumulated number of laser pulses. The average ranging error obtained from 10 simulated measurements is shown in Figure 12. Figure 12a–h illustrates that as the *H* increases, there is a gradual increase in the ranging error obtained by various methods. In particular, the photon counting entropy, matched filter, direct thresholding, Frank–Wolfe, proximal gradient, and wavelet threshold methods exhibit a significant increase in ranging error, reaching over 200 cm. The proposed method demonstrates superior ranging performance, with the error within 20 cm in Figure 12c and within 10 cm for the others.

### 5.2. Experimental Analysis

**Figure 13:**
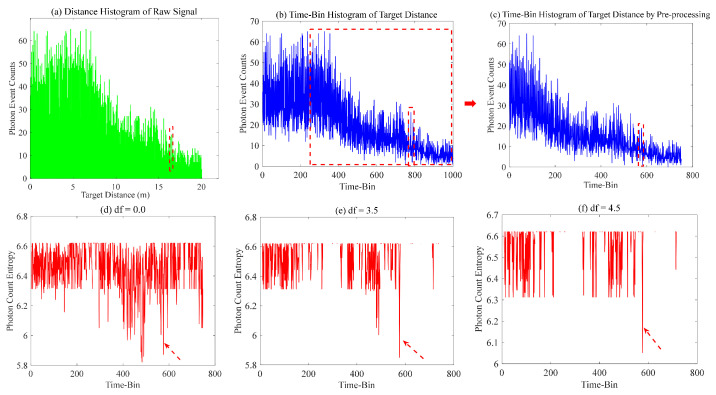
The analysis procedure of photon counting entropy.

The initial test of our proposed method involved conducting the ranging experiment at a target distance of 16 m. The distance histogram of PEs corresponding to the observed data was obtained and is displayed in Figure 13a. The time-bin histogram can be generated within the specified range of 0–20 m by selecting a distance resolution of 0.02 m, as depicted in Figure 13b. The general signal is typically located in the descending region of the histogram. The primary cause of the initial rise zone is mainly attributed to the interference caused by solar background noise. The drop region (5–20 m) can be preserved by truncating the observed data during pre-processing, as shown in Figure 13c. Various distinct values (df = 0.0, 3.5, and 4.5) of offset parameter can result in different outcomes for the photon counting entropy, as illustrated in Figure 13d–f. The determination of the target signal’s location becomes challenging in Figure 13d due to the presence of multiple peaks caused by not optimizing the offset parameter (df = 0.0), which is a comparison method based on the photon counting entropy. The peaks caused by solar background noise can be effectively eliminated by setting df = 4.5, and the corresponding target position can be clearly distinguished.

**Figure 14:**
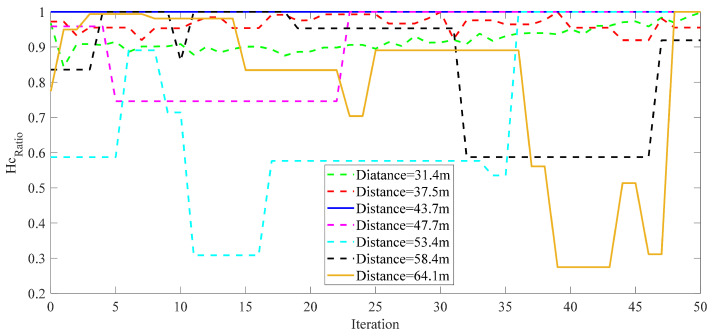
The threshold determination of $Hc_{ratio}$.

The optimal offset parameter df is determined using our proposed method based on the desired threshold condition for $Hc_{ratio}$. By selecting seven groups of distances, with *K* = $5\times 10^{4}$, and conducting 50 iterations to calculate the corresponding $Hc_{ratio}$, the threshold is determined, as depicted in Figure 14. The figure illustrates that the majority of values are concentrated above 0.6. Therefore, we choose 0.6 as the desired $Hc_{ratio}$ threshold.

The pre-processing procedure involved truncating the ascending region of the initial 20 m range and retaining only the descending region where the target signal was present. Through the prediction of the MLP network and linear conversion in Figure 7b, the noise rates corresponding to distances of 31.4, 37.5, 43.7, 47.7, 53.4, 58.4, and 64.1 m were estimated as follows: 12.26, 12.09, 12.53, 12.51, 12.72, 12.96, and 12.38 MHz. The effectiveness of our proposed method was validated through three aspects, namely, the accumulated number of laser pulses, attenuation angle, and target distance.

**Figure 15:**
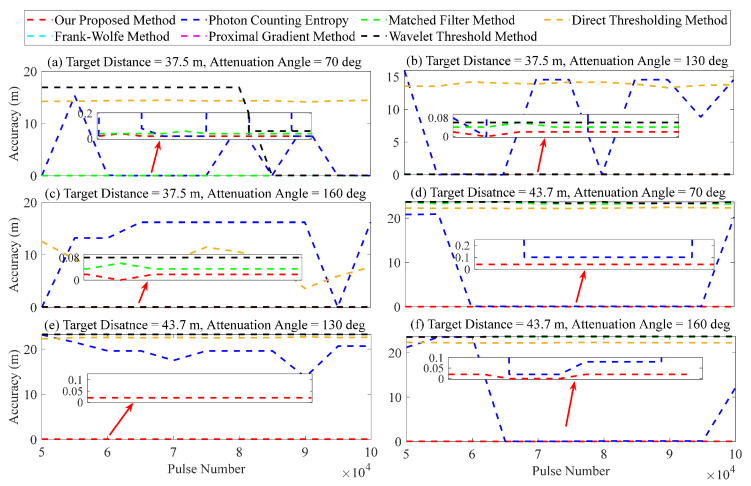
Ranging error of the different accumulated numbers of laser pulses.

First, for the accumulated number *K* of laser pulses, the performance of the proposed method was validated by the following parameters: The values of *H* were selected as 37.5 and 43.7 m; the attenuation angles were 70, 130, and 160 deg; the SNR varied between 0.47 and 7.17; and *K* ranged from $5\times 10^{4}$ to $1\times 10^{5}$, with an interval of $0.5\times 10^{4}$. The results are presented in Figure 15.

Figure 15 demonstrates that the matched filter, Frank–Wolfe, proximal gradient, and wavelet threshold methods exhibit high sensitivity to the intensity of the target signal for lidar. The ranging error of these methods can exceed 20 m when the target is located at a greater distance (43.7 m) and the signal strength is weak, as depicted in Figure 15d–f. The ranging error remains small, less than 20 cm, when the target distance is 37.5 m and the signal strength is high, as depicted in Figure 15a–c. The direct thresholding method in Figure 15 may exhibit sensitivity to the signal strength of two target distances, potentially resulting in a range error of up to 20 m. The method for measuring photon counting entropy shows significant fluctuations. In favorable scenarios, the ranging error is less than 10 cm. However, in unfavorable scenarios, it can exceed 40 m, indicating a lack of stability. Compared to other methods, our proposed method demonstrates superior accuracy in error within a range of 5 cm in Figure 15.

**Figure 16:**
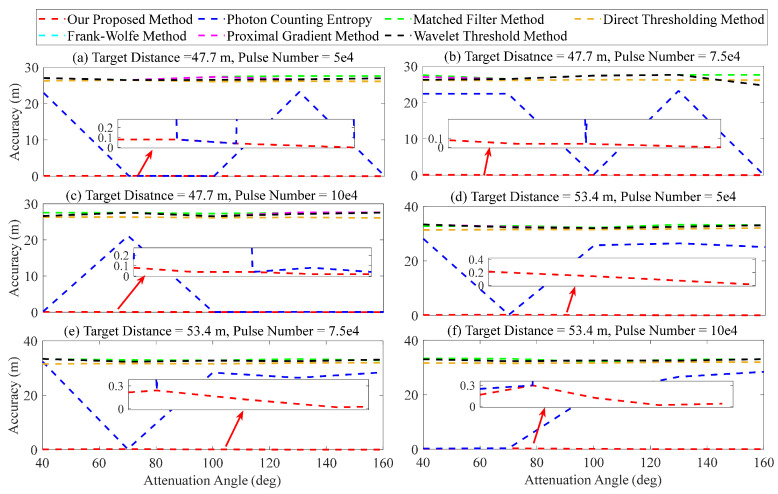
Ranging error of different attenuation angles.

Furthermore, in terms of attenuation angle, the superiority of our proposed method is validated through comparison with other methods. We selected two target distances (47.7 and 53.4 m), three accumulated numbers ($5\times 10^{4}$, $5\times 7.5^{4}$, and $1\times 10^{5}$), and five attenuation angles (40, 70, 100, 130, and 160 deg) for analysis. The corresponding SNR varied between 1.03 to 2.10. The results are presented in Figure 16. Figure 16a–f demonstrates that the matched filter, direct thresholding, Frank–Wolfe, proximal gradient, and wavelet threshold methods yield significant range error when analyzing two target distances (47.7 and 53.4 m) due to the high sensitivity of the strength of target signal. The error can exceed 30 m, which poses challenges in accurately determining the target’s location. The method of photon counting entropy demonstrates significant fluctuations, as evidenced by Figure 16a–f. For example, in Figure 16a, the ranging error is approximately 10 cm when the attenuation angle is 70 and 100 deg, whereas it increases to about 23 m at an attenuation angle of 130 deg. Our proposed method demonstrates excellent performance of signal recognition, with the error ranging from 10 to 30 cm.

**Figure 17:**
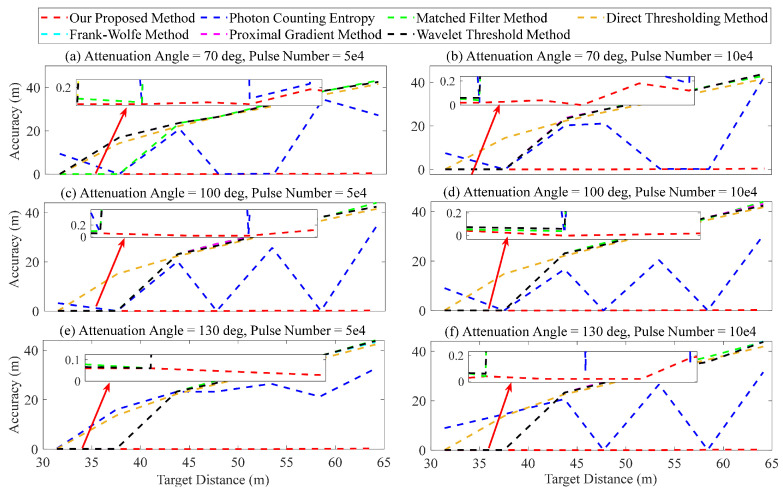
Ranging error at different target distances.

Finally, the performance of the proposed method was further validated by comparing it with other methods in terms of various target distances. We utilized three attenuation angles (70, 100, and 130 deg), two accumulated numbers ($5\times 10^{4}$ and $10\times 10^{5}$) of laser pulses, and seven target distances (31.4, 37.5, 43.7, 47.7, 53.4, 58.4, and 64.1 m) for analysis. The calculated SNR ranged from 0.43 to 17.84. The error outcomes are presented in Figure 17. As shown in Figure 17a–f, there is a gradual decrease in signal strength as the target distance increases. The ranging error, calculated using the matched filter, direct thresholding, Frank–Wolfe, proximal gradient, and wavelet threshold methods, exhibits an upward trend and eventually reaches 40 m. Consequently, accurately determining the target’s location becomes challenging. The ranging error, calculated using the method of photon counting entropy, as shown in Figure 17a–f, also exhibits significant fluctuations. For example, the ranging error in Figure 17a is approximately 20 cm when the target distance is 37.5 m, but it increases to about 20 m at a target distance of 43.7 m. Compared with other methods, our proposed method demonstrates superior recognition performance, with the error within 30 cm.

## 6. Conclusions

The primary focus of our research is to address the issue of significant fluctuations in ranging error caused by the presence of multiple peaks in photon counting entropy. Under the framework of photon counting entropy, we propose a denoising algorithm for optimizing df, including two main aspects. Firstly, by utilizing the MLP network for prediction and linear conversion, a more accurate estimation of the ${\hat{\lambda }}_{n}\left(t\right)$ in the solar background can be achieved, thereby establishing a solid foundation for reducing noise. Subsequently, we propose an offset parameter df optimization algorithm to effectively eliminate peak interference caused by noise, and enhance the performance of location recognition for the target signal.

To validate the performance of our proposed method, we analyze the ranging error of lidar in simulations and experiment. The simulation part involves analyzing three key factors, namely, noise rate, the accumulated number of laser pulses, and target distance, and the proposed method exhibits a ranging error of approximately 5 cm. Compared with other methods (photon counting entropy, matched filter, direct thresholding, Frank–Wolfe, proximal gradient, and wavelet threshold methods), the average ranging error is enhanced by 81.99%, 76.98%, 83.44%, 81.85%, 81.85%, and 81.60%, respectively. The experimental part considers three factors (namely, the accumulated number of radar pulse, attenuation angle, and target distance), and the ranging error calculated by our proposed method is within 30 cm. The average ranging error is also increased by 73.76%, 86.29%, 97.26%, 90.81%, 90.80%, and 90.80%, respectively, when compared to the other six methods.

## Figures and Tables

**Table 1:** Size distribution parameters for rain and fog.

Distribution	*a*	*b*	*c*	*d*
Heavy fog	0.027	0.3	3	1
Moderate fog	607.5	3	6	1
Chu and Hogg fog	341	4	2	0.5
Haze H	$4.0000\times 10^{5}$	20	2	1
Haze L	$4.9757\times 10^{6}$	15.1186	2	0.5
Haze M	$5.3333\times 10^{4}$	8.9443	1	0.5
Rain L	$4.9757\times 10^{7}$	15.1186	2	0.5
Rain M	$5.3333\times 10^{5}$	8.9443	1	0.5

**Table 2:** The transmittance (τ) values of various weather conditions and propagation distances.

Size Distribution F(r)								
*H* (m)	Heavy Fog	Moderate Fog	Chu and Hogg Fog	Haze H	Haze L	Haze M	Rain L	Rain M
100.0	0.7783	0.4219	0.8747	0.9923	0.9946	0.9845	0.9451	0.8941
150.0	0.6867	0.2741	0.8181	0.9884	0.9919	0.9769	0.9188	0.8455
200.0	0.6058	0.1780	0.7651	0.9846	0.9892	0.9693	0.8932	0.7994
250.0	0.5345	0.1156	0.7156	0.9808	0.9865	0.9617	0.8683	0.7559
300.0	0.4715	0.0751	0.6693	0.9770	0.9838	0.9543	0.8442	0.7148
350.0	0.4160	0.0488	0.6260	0.9732	0.9811	0.9468	0.8207	0.6759
400.0	0.3670	0.0317	0.5854	0.9694	0.9785	0.9395	0.7978	0.6391
450.0	0.3238	0.0206	0.5476	0.9657	0.9758	0.9322	0.7756	0.6043
500.0	0.2857	0.0134	0.5121	0.9619	0.9732	0.9249	0.7540	0.5714
550.0	0.2520	0.0087	0.4790	0.9582	0.9705	0.9178	0.7330	0.5403
600.0	0.2223	0.0056	0.4480	0.9545	0.9679	0.9106	0.7126	0.5109
650.0	0.1962	0.0037	0.4190	0.9508	0.9653	0.9035	0.6928	0.4831
700.0	0.1731	0.0024	0.3918	0.9471	0.9626	0.8965	0.6735	0.4568
750.0	0.1527	0.0015	0.3665	0.9435	0.9600	0.8896	0.6547	0.4320
800.0	0.1347	0.0010	0.3428	0.9398	0.9574	0.8826	0.6365	0.4085
850.0	0.1188	0.0007	0.3206	0.9362	0.9548	0.8758	0.6188	0.3862
900.0	0.1048	0.0004	0.2998	0.9325	0.9522	0.8690	0.6016	0.3652
950.0	0.0925	0.0003	0.2804	0.9289	0.9496	0.8622	0.5848	0.3453
1000.0	0.0816	0.0002	0.2623	0.9253	0.9470	0.8555	0.5685	0.3265

**Table 3:** The hyperparameter values for the MLP model.

Model	Model Hyperparameter	Value
MLP	Dense 1	128
	Dropout 1	0.33
	Dense 2	64
	Dropout 2	0.33
	Dense 3	32
	Dropout 3	0.33
	Dense 4	1
	Learn rate	0.001
	Epochs	30
	Batch size	32

**Table 4:** Simulation parameters for the single-photon-counting lidar.

Module	Parameter	Value
Laser	Wavelength	532 nm
	Repetition period	4 kHz
	Pulse width	0.32 ns
	Single-shot energy	5 nJ
Detector	Quantum efficiency	0.55
	Dead time	45 ns
Receiving system	Aperture	5 cm
TCSPC	Time resolution	0.13 ns
Target	Size	21 × 29.7 ${\mathrm{cm}}^{2}$

**Table 5:** Power meter specifications.

Model	843-R-USB Power Meter
Dimension (W × D × H )	213 × 113 × 40 mm
Mass	0.47 kg
Sampling Frequency	Photodiode and thermopile: 15 Hz
	Pyroelectric: 500 Hz
Resolution Ratio	18 bits plus sign
Accuracy	±0.25 (full scale) ± 20 PA

**Table 6:** Thermopile sensor specifications.

Model	919P-003-10 Thermopile Sensor
Maximum Measured Power	3 W
Spectral Region	0.19∼11 μm
Effective Diameter	9.5 mm

**Table 7:** Anemometer specifications.

Measuring Terms	Measurement Range	Accuracy
Temperature (°C)	−20.0∼60.0	±1.0
Relative Humidity (%)	0∼100.0	±5
Pressure (hPa)	300∼1100	±0.1
Dew Point (°C)	−40.0∼60.0	±2.0
Wind Speed (m/s)	0.7∼30.0	±0.3
Altitude (m)	−500∼9000	-

**Table 8:** Description of meteorological variables for predicting GHI.

Repository	Variable	Description	Units
Experiment	GHI	Global Horizontal Irradiance	$W/m^{2}$
	DNI	Direct Normal Irradiance	$W/m^{2}$
	DHI	Diffuse Horizontal Irradiance	$W/m^{2}$
	RH	Relative Humidity	%
	DP	Dew Point	°C
	T	Temperature	°C
	MWS	Meridional Wind Speed	m/s
	LWS	Latitudinal Wind Speed	m/s
	P	Pressure	hPa
	A	Altitude	m

**Table 9:** The descriptive statistics of the GHI.

Time	May 2023–January 2024
Interval time	5 min
Count	276
Median	397.4 $W/m^{2}$
Mean	413.8 $W/m^{2}$
Standard deviation	263.1 $W/m^{2}$
Maximum	877.5 $W/m^{2}$
Minimum	10.1 $W/m^{2}$

## Data Availability

Data available on request due to privacy.

## Appendix A. Validity Verification of Photon Counting Entropy

If it is a pure signal, the random fluctuation of the detected count $\hat{y}\left(i\right)$ corresponding to the *i*-th bin can be represented by a Gaussian function.

(A1) $$\hat{y}\left(i\right)=\frac{N_{s}}{\sqrt{2\pi }\sigma_{s}}exp\left(-\frac{i^{2}{\Delta t}^{2}}{2{\sigma_{s}}^{2}}\right)$$

The Fourier transform is applied and then summed, resulting in Equations (A2) and (A3).

(A2) $$\begin{array}{cc} g(i) & =\hat{y}\left(i\right)exp\left(-2\pi kij/N_{k}\right)\\ \; & =\frac{N_{s}}{\sqrt{2\pi }\sigma_{s}}exp\left(-\frac{2{\sigma_{s}}^{2}\pi^{2}k^{2}}{{\Delta t}^{2}{N_{k}}^{2}}\right)exp\left(-\frac{{\Delta t}^{2}}{2{\sigma_{s}}^{2}}\left(1+\frac{2{\sigma_{s}}^{2}j\pi k}{{\Delta t}^{2}}N_{k}\right)\right) \end{array}$$

(A3) $$\begin{array}{c} \hat{Y}\left(k\right)=2\sum_{i=1}^{N}g\left(i\right)=\frac{N_{s}}{\Delta t}exp\left(-\frac{2{\sigma_{s}}^{2}\pi^{2}k^{2}}{{\left(\Delta t\right)}^{2}{N_{k}}^{2}}\right) \end{array}$$

The value of $\hat{Y}\left(k\right)$ is squared and then the modulus is taken.

(A4) $${|\hat{Y}\left(k\right)|}^{2}=\frac{{N_{s}}^{2}}{{\left(\Delta t\right)}^{2}}exp\left(-\frac{4{\sigma_{s}}^{2}\pi^{2}k^{2}}{{\left(\Delta t\right)}^{2}{N_{k}}^{2}}\right)$$

The photon counting entropy can be obtained by summing and applying the logarithm, resulting in the following expression.

(A5) $$X_{k}=\frac{{|\hat{Y}\left(k\right)|}^{2}}{\sum_{k=1}^{N_{k}}{|\hat{Y}\left(k\right)|}^{2}}=\frac{4\sigma_{s}\sqrt{\pi }}{{\left(\Delta t\right)}^{2}N_{k}}exp\left(-\frac{4{\sigma_{s}}^{2}\pi^{2}k^{2}}{{\left(\Delta t\right)}^{2}{N_{k}}^{2}}\right)$$

(A6) $$\begin{array}{cc} Z_{k} & =X_{k}ln\left(\frac{1}{X_{k}}\right)\\ \; & =\frac{4\sigma_{s}\sqrt{\pi }}{{\left(\Delta t\right)}^{2}N_{k}}exp\left(-\frac{4{\sigma_{s}}^{2}\pi^{2}k^{2}}{{\left(\Delta t\right)}^{2}{N_{k}}^{2}}\right)\left(ln\left(\frac{\Delta tN_{k}}{4\sigma_{s}\sqrt{\pi }}\right)+\frac{4{\sigma_{s}}^{2}\pi^{2}k^{2}}{{\left(\Delta t\right)}^{2}{N_{k}}^{2}}\right) \end{array}$$

(A7) $$H_{c\_signal}=\sum_{k=1}^{N_{k}}Z_{k}=ln\left(\frac{\sqrt{e}N_{k}}{4\sqrt{\pi }\Delta \sigma_{s}}\right)$$

where $\Delta \sigma_{s}=\sigma_{s}/\Delta t$ represents the standard deviation of the laser pulse.

The noise spectral distribution characteristics are typically uniform when it is pure noise.

(A8) $$\hat{Y}\left(k\right)=const$$

The photon counting entropy of the noise can be calculated as follows.

(A9) $$X_{k}=\frac{{|\hat{Y}\left(k\right)|}^{2}}{\sum_{k=1}^{N_{k}}{|\hat{Y}\left(k\right)|}^{2}}=\frac{1}{N_{k}}$$

(A10) $$Z_{k}=X_{k}ln\left(\frac{1}{X_{k}}\right)=\frac{ln(N_{k})}{N_{k}}$$

(A11) $$H_{c\_noise}=\sum_{k=1}^{N_{k}}Z_{k}=ln\left(N_{k}\right)$$

The photon counting entropy of the signal is lower than that of the noise because $\Delta \sigma_{s}$ significantly exceeds 1, resulting in $\frac{\sqrt{e}N_{k}}{4\sqrt{\pi }\Delta \sigma_{s}}$ being less than $N_{k}$.
